# Altered Intra- and Inter-regional Functional Connectivity of the Anterior Cingulate Gyrus in Patients With Tremor-Dominant Parkinson’s Disease Complicated With Sleep Disorder

**DOI:** 10.3389/fnagi.2019.00319

**Published:** 2019-11-21

**Authors:** Xinjie Chen, Xiaoyan Hou, Xiaodong Luo, Sifan Zhou, Xian Liu, Bo Liu, Jun Chen

**Affiliations:** ^1^The Second Affiliated Hospital of Guangzhou University of Chinese Medicine, Guangzhou, China; ^2^Department of Radiology, Guangdong Hospital of Traditional Chinese Medicine, Guangzhou, China; ^3^Department of Radiology, ZHUHAI Branch of Guangdong Hospital of Traditional Chinese Medicine, Zhuhai, China

**Keywords:** tremor-dominant Parkinson’s disease, sleep disorder, regional homogeneity, functional connectivity, anterior cingulate gyrus

## Abstract

**Objective**: To investigate changes in brain function at the regional and whole-brain levels in patients with tremor-dominant Parkinson’s disease (TDPD) complicated by sleep disorder (SD) by regional homogeneity (ReHo) and functional connectivity (FC) analysis of whole-brain resting-state functional magnetic resonance images.

**Materials and Methods**: ReHo and seed-based FC analyses were conducted among 32 patients with TDPD and SD (TDPD-SD), 24 with TDPD and no SD (TDPD-NSD), and 23 healthy controls (HCs) to assess spontaneous brain activity and network-level brain function. Correlation analyses were used to examine the associations between brain activity and the clinical data.

**Results**: Anterior cingulate gyrus (ACC) ReHo values differed significantly among the groups. ACC ReHo values were increased in TDPD-SD vs. HC and TDPD-SD vs. TDPD-NSD. ACC ReHo values were reduced in TDPD-NSD vs. HC. TDPD-SD ReHo values were positively correlated with Pittsburgh Sleep Quality Index (PSQI) scores (*r* = 0.41, *p* = 0.020) but negatively correlated with Parkinson’s Disease Sleep Scale (PDSS) scores (*r* = −0.38, *p* = 0.030). FC analysis using ACC as a mask showed that FC of the left olfactory cortex (L-OC), right straight gyrus (R-SG), right superior parietal gyrus (R-SPG), and right precuneus differed significantly among the groups. FC values between R-SG and ACC were significantly lower in TDPD-SD than in TDPD-NSD, while the FC of L-OC and R-OC with ACC was significantly lower in TDPD-SD than in HC. FC between ACC and L-OC, R-SPG, and the right precuneus was lower in TDPD-NSD than in HC. There was no correlation between the FC values and other clinical data in any of the groups.

**Conclusion**: Localized abnormal activity in TDPD-SD was chiefly triggered by ACC. The change in the ReHo of ACC is closely related to the severity of TDPD-associated SD, revealing the role of this region as a regulator of the sleep mechanism in TDPD. Significant abnormal FC was found between R-SG and ACC in TDPD-SD but was not shown to correlate with clinical data.

## Introduction

Parkinson’s disease (PD) is a degenerative neurological disorder characterized by extrapyramidal dyskinesia with a variety of accompanying non-motor symptoms (Jankovic, 2008). Sleep disorder (SD) is a common non-motor symptom of PD, with an incidence of approximately 60–98% in PD patients (Kumar et al., 2002). The etiology and pathogenesis of PD-associated SD are complicated. Current data suggest that the causes of PD-associated SD are multifactorial and may largely overlap with triggers for idiopathic SD (Kumar et al., 2002), while the pathophysiology of PD can also indirectly impair patients’ sleep cycles by increasing their susceptibility to sleep disturbances (Albers et al., 2017).

Previous findings suggest that PD-associated SD may be caused by abnormal neural activity (Askenasy, 1993). It is generally believed that the loss of dopaminergic neurons in the nigra causes an imbalance of neurotransmitter activity in the brain, further leading to the loss and damage of neural circuits and altering the structure of sleep centers and the function of neurotransmitters (Comella, 2007). In addition to the disease itself, anti-Parkinson’s drugs and other related psychiatric symptoms can further affect sleep-related brain neural activity (Videnovic and Golombek, 2013). However, the neural mechanism of PD-associated SD, especially in terms of brain pathophysiology, has not been clarified thus far. Previous studies have examined magnetic resonance imaging structural changes (cortical thickness, cortical and subcortical volume) in PD patients with SD (Radziunas et al., 2018). In recent years, studies have started to use functional magnetic resonance imaging (fMRI) to study rapid eye movement sleep behavior disorder (RBD), a subtype of PD-associated SD (Li et al., 2017), and found abnormal changes in neural signals in the caudate, putamen, and prefrontal cortex (PFC) in PD patients with RBD.

While the abovementioned studies have provided essential information to unravel the neural correlates of PD-associated SD, current knowledge of the mechanisms underlying PD-associated SD remains limited. In particular, there is some fMRI evidence that has revealed significant differences in neural circuits between tremor-dominant PD (TDPD) and non-tremor-dominant PD (NTDPD; Spiegel et al., 2007). Tremors are one of the main motor symptoms of PD, and 70% of clinical PD cases display significant resting-state tremors and/or postural tremors (He et al., 2015). Previous studies have shown characteristic changes in the neural activity of the basal ganglia, limbic system, cerebellum, and other brain regions in patients with TDPD (Rivlin-Etzion et al., 2010). Autopsies and animal studies related to PD have also suggested a discrepancy between the neural pathways of TDPD and NTDPD (Mounayar et al., 2007). Thus, compared with NTDPD, TDPD has obvious abnormal neural activity, which may further exert different neurophysiological effects on the neural mechanism of nonmotor symptoms of PD. There have been no additional studies on the brain abnormal neural activity of SD in a single motor subtype of PD.

Therefore, based on previous studies that have discovered abnormal brain neural activity in TDPD and PD-associated SD, we further hypothesized that TDPD-SD may feature unique brain abnormalities at the neurophysiological level, showing a characteristic pattern of brain neural activity. In this study, a resting-state fMRI (rs-fMRI) method was used to search for evidence to support our hypothesis, which can monitor the pathological and physiological changes in the brains of PD patients (Huettel et al., 2004). Regional homogeneity (ReHo) analysis and seed-based functional connectivity (FC) are two main approaches to analyze rs-fMRI data (Greicius et al., 2003; Zang et al., 2004), which were performed in this study. To date, ReHo and FC analyses have been applied to many aspects of PD-related fMRI studies, including localized changes in brain functions that are manifested by symptoms such as fatigue (Li et al., 2017), depression (Wen et al., 2013), and cognitive decline (Harrington et al., 2017). To date, however, no research has applied the methods of ReHo and FC analysis in PD-associated SD.

This study will further explore the uniqueness and abnormality of brain region synergy and the brain neural network in TDPD-SD to further test the experimental hypothesis.

## Materials and Methods

### Participants

This study was conducted at the Second Affiliated Hospital of Guangzhou University of Chinese Medicine between May 2018 and December 2018. A total of 56 TDPD patients who had been diagnosed and treated in the Chronic PD Clinic were selected for this study. The subjects included 33 male and 23 female patients between 50 and 83 years of age, with a mean age of 64.4 ± 6.9 years. The following inclusion criteria were used: (1) all patients met the Parkinson’s diagnostic criteria of the UK Brain Bank and exhibited symptoms consistent with the clinical classification of TDPD; (2) the patients ranged in age from 50 to 83 years and had at least 5 years of formal education; (3) PD was graded as ≤III by the Hoehn & Yahr scale (H & Y); and (4) all patients were right handed. The following exclusion criteria were used: (1) patients with secondary Parkinson’s syndrome or Parkinson’s superposition syndrome; (2) patients with severe heart, liver, or kidney disease, hematopoietic disorders, or malignant tumors; (3) patients <40 or >85 years old; and (4) patients who did not undergo fMRI for various reasons. All enrolled patients were required to complete a series of evaluations including the Movement Disorder Society-Sponsored Revision Unified Parkinson’s Disease Rating Scale (MDS-UPDRS), the Hamilton Depression Rating Scale (HAM-D), the Parkinson’s Disease Sleep Scale (PDSS), and the Pittsburgh Sleep Quality Index (PSQI). A total of 32 PD patients with SD symptoms were included in the TDPD-SD group, and 24 patients who had no SD symptoms were included in the TDPD-NSD group. Healthy controls (HCs; *n* = 23) were enrolled to form a group composed of 11 male and 12 female patients aged 50–82 years with an average age of 61.4 ± 8.5 years. All individuals in the control group completed the Mini-Mental State Examination (MMSE) and the HAM-D assessment. This study was approved by the Ethics Committee of the Second Affiliated Hospital of Guangzhou University of Chinese Medicine, and all subjects provided written informed consent.

### Image Acquisition

All participants underwent structural MRI and rs-fMRI scans on a 3.0-T MR system (Magnetom Verio, Siemens, Germany) at the Department of Radiology at the Second Affiliated Hospital of Guangzhou University of Chinese Medicine. To eliminate the effect of levodopa on the brain, the fMRI scan was performed at least 4 h after levodopa administration. Participants in the TDPD-SD group who regularly took sleep aids were informed to stop taking those medications for at least 24 h before the fMRI scans. Cushions were used to support participants’ heads within the coil to prevent excessive head motion. To prevent any active or passive movements of the head or body, the subjects were instructed to close their eyes and breathe calmly while remaining still. All images were acquired in parallel to the anterior–posterior commissure line with an auto alignment technique. The functional data were collected using an echo-planar imaging sequence with the following parameters: 31 axial slices; repetition time = 2,000 ms; echo time = 30 ms; slice thickness = 3.5 mm; gap = 0.7 mm; flip angle = 90°; matrix = 64 × 64; field of view = 224 mm × 224 mm; and 240 time points. 3D structural images were acquired using a T1-weighted magnetization-prepared rapid gradient-echo sequence with the following parameters: 176 sagittal slices; repetition time = 1,900 ms; echo time = 2.3 ms; inversion time = 900 ms; slice thickness = 1.0 mm, no gap; flip angle = 9°; matrix = 256 × 256; and field of view = 256 mm × 256 mm.

### Preprocessing of rs-fMRI Data

The MATLAB-based programs DPABI V3.1^1^ (Yan et al., 2016) and SPM12^2^ were used for preprocessing of the rs-fMRI data analysis. The first 10 time points of each subject were discarded to allow the signal to reach equilibrium and the participant to adapt to the scanning noise. Then, realignment was performed to correct for head motion between time points. All functional data were within the defined motion thresholds (i.e., translational or rotational motion parameters <3 mm or 3°). Immediately following the realignment, the spatial normalization of fMRI images was performed. During spatial normalization, individual structural images were first coregistered with the mean functional images derived from data after correcting for head motions. The registered structural images were then segmented and normalized to the Montreal Neurological Institute (MNI) space using a high-level nonlinear warping algorithm, that is, diffeomorphic anatomical registration through exponentiated Lie algebra (DARTEL; Calhoun et al., 2017). In this step, individual T1 structural images were segmented (the segments included white matter, gray matter, and cerebrospinal fluid) using the DARTEL segment toolbox based on DPABI. Next, each structural image was coregistered to the functional image and then reconstructed into 3 × 3 × 3 mm resolution voxels. Then, a specific study template was created using the DARTEL toolkit to transform the individual native space to standard MNI space. Then, the resulting fMRI data were bandpass filtered (0.01–0.08 Hz), and the linear trend of time courses was removed. Covariates and their temporal derivatives were then removed from the data by linear regression analysis to minimize impacts of cardiac effects and respiratory, including the global signal, white matter, cerebrospinal fluid, and Friston’s 24 parameters of head motion (including six head motion parameters, the same six head motion parameters one time point before, and the 12 corresponding squared items). Spatial smoothing was performed in preprocessing of FC analysis but not ReHo analysis, as this step would have affected the result of local ReHo measurement and reduced its reliability.

### ReHo Analysis

ReHo calculation was performed using Kendall’s coefficient of concordance (KCC) to measure the synchronicity of the time series between a given voxel with its 26 nearest neighbors in a voxelwise way. To reduce the influence of individual variations in the KCC value, normalization of ReHo maps was performed by dividing the KCC among each voxel by the averaged ReHo of the whole brain. Finally, the ReHo maps were smoothed using a 6-mm full width at half maximum Gaussian kernel to reduce the noise and residual differences in the gyral anatomy.

### Seed-Based FC Analysis

Regions showing significant differences in ReHo values among the three groups, as revealed by one-way analysis of variance (ANOVA), were defined as regions of interests (ROIs) for seed-based FC analysis to investigate interregional functional synchronization. ROIs were extracted from the ROIs using xjView based on SPM12 (Yan et al., 2016), with a cluster-level threshold of *p* < 0.05 (familywise error, FWE-corrected) and a cluster-forming threshold at voxel level *p* < 0.001 as mask. According to the results, the bilateral anterior cingulate gyrus (ACC) was chosen as the ROI. Specifically, the mean time course for each ROI was extracted and correlated (by Pearson correlation) with time series of each voxel over the whole brain for each participant. Subsequently, the resultant correlation values were subjected to a Fisher *r*-to-*z* transformation to improve normality.

### Statistical Analysis

Differences in demographic, clinical, and cognitive variables: ANOVA was conducted to test differences in age among the three groups, while the Kruskal–Wallis test was conducted to test differences in MMSE and HAM-D scores among the three groups. The differences in the sex distribution among the three groups were examined with a chi-squared test. The independent two-sample *t*-test was used to compare PSQI, PDSS, MDS-UPDRS, and MDS-UPDRS-III scores; durations of PD; dosages of levodopa tablets; and H & Y stages between the TDPD-SD and TDPD-NSD groups. Differences were considered statistically significant when *p* < 0.05.

#### Differences in ReHo and FC Analyses

Voxelwise ANOVA analyses of ReHo and FC were performed among the three groups, with age as a covariate. Two-sample tests were conducted within the mask obtained from the ROI result of ANOVA in the *post hoc* analysis of ReHo and FC. Statistical significances were corrected by a cluster-level threshold of *p* < 0.05 (FWE-corrected) and a cluster-forming threshold at voxel level *p* < 0.001 in ANOVA and *post hoc* analyses of ReHo analysis. In the FC analysis, statistical significance was corrected by a cluster-level threshold of *p* < 0.05 (FWE-corrected) and a cluster-forming threshold at voxel level *p* < 0.005 in ANOVA, and a cluster-level threshold of *p* < 0.05 (FWE-corrected) and a cluster-forming threshold at voxel level *p* < 0.001 in the *post hoc* analyses.

### Correlation Analysis

To determine the relationship between imaging and clinical data in the TDPD-SD group, Pearson’s correlation analysis was conducted. Spearman’s correlation analysis was used to compare part of the clinical data (MMSE scores, HAM-D scores) and ReHo values of patients in the PDSD-associated/NSD group. A threshold of *p* < 0.05 was considered statistically significant.

## Results

### Differences in Clinical Data

There were no significant differences in age, sex, HAM-D scores, or MMSE scores among the three groups (all *p* > 0.05). There were no significant differences in MDS-UPDRS and MDS-UPDRS-III scores, H–Y grades, disease duration, or daily doses of levodopa tablets between the two patient groups (all *p* > 0.05). Compared with the TDPD-NSD group, the TDPD-SD group exhibited significantly higher PSQI scores (*t* = −16.55, *p* < 0.05) and lower PDSS scores (*t* = 11.04, *p* < 0.05; see Table 1).

**Table 1:** Demographic and clinical characteristics of 56 TDPD patients and 23 healthy controls.

	TDPD-SD	TDPD-NSD	HC	*F/χ^2^/H/t*	*p*-value
Age	65.50 (7.73)	63.04 (5.65)	61.43 (8.46)	2.12	0.13^a^
Gender (male, %)	42.86%	75.00%	52.17%	5.55	0.062^b^
Education	11.28 (3.12)	11.46 (3.41)	12.72 (2.71)	1.556	0.22^a^
HAM-D	8.59 (4.25)	7.08 (5.08)	6.22 (2.33)	2.39	0.099^c^
MMSE	27.38 (3.15)	27.38 (2.53)	26.26 (2.14)	1.38	0.26^c^
PSQI	11.50 (1.39)	1.63 (0.88)		32.48	0.037^d^
PDSS	110.19 (16.30)	129.54 (11.62)		−4.66	0.039^d^
H & Y	1.91 (0.50)	2.04 (0.62)		−0.69	0.49^d^
Disease duration (years)	5.44 (3.88)	5.17 (3.91)		−0.14	0.89^d^
MDS-UPDRS	49.66 (21.83)	44.71 (20.77)		−0.70	0.48^d^
MDS-UPDRS III	28.50 (16.12)	25.79 (13.36)		−0.44	0.66^d^

*TDPD-SD, tremor-dominant Parkinson’s disease with sleep disorder; TDPD-NSD, tremor-dominant Parkinson’s disease with no sleep disorder; HC, healthy controls; HAM-D, Hamilton Depression Rating Scale; MMSE, Mini-Mental State Examination; H & Y, Hoehn & Yahr scale; PSQI, Pittsburgh Sleep Quality Index; PDSS, Parkinson’s Disease Sleep Scale; MDS-UPDRS, Unified Parkinson’s Disease Rating Scale. Values are given as the mean (SD) except for sex, which is presented as the proportion of male patients. Group differences were tested using SPSS software. Differences were considered statistically significant when *p* < 0.05. ^a^*p*-value obtained with one-way analysis of variance. The test statistic of the method is called the *F*-statistic. ^b^*p*-value obtained with chi-squared test. The test statistic of the method is called the *χ*^2^ statistic. ^c^*p*-value obtained with the Kruskal–Wallis test. The test statistic of the method is called the *H* statistic. ^d^*p*-value obtained with independent *t*-test for continuous variables. The test statistic of the method is called the *t*-statistic*.

### ReHo Analysis Results

**Figure 1:**
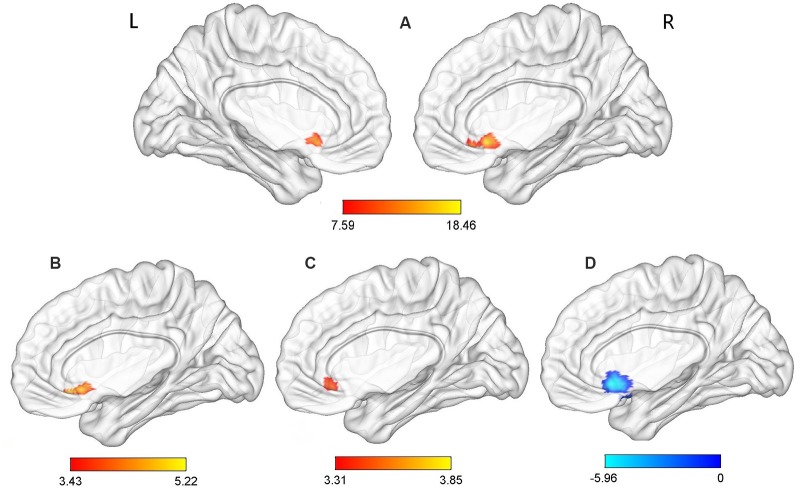
Analysis of variance (ANOVA) and *post hoc* analysis results for regional homogeneity (ReHo). **(A)** ANOVA among three groups; **(B)** comparison between the tremor-dominant Parkinson’s disease with no sleep disorder (TDPD-NSD) and with sleep disorder (TDPD-SD) groups; **(C)** comparison between the healthy control (HC) and TDPD-SD groups; **(D)** comparison between the TDPD-NSD and HC groups; color bars indicate the *t*-score, with warm-colored areas representing brain regions where ReHo/functional connectivity (FC) values increased and cool-colored areas representing brain regions where ReHo/FC values decreased. R, right; L, left.

Significant group effects were observed for the ReHo values in the ACC (*p*-voxel < 0.001, *p*-cluster correction < 0.05). Further *post hoc* comparisons revealed the following: (1) compared with the TDPD-NSD group, TDPD-SD exhibited significantly higher ReHo values in ACC (*p*-voxel < 0.001, *p*-cluster correction < 0.05); (2) compared with the HC group, TDPD-SD exhibited significantly higher ReHo values in ACC (*p*-voxel < 0.001, *p*-cluster correction < 0.05); and (3) compared with the HC group, TDPD-NSD exhibited significantly lower ReHo values in ACC (*p*-voxel < 0.001, *p*-cluster correction < 0.05; see Table 2, Figure 1).

**Table 2:** Brain areas with ReHo and FC differences among all groups.

Region (AAL)	Side	Voxels	Peak	MNI coordinate (*x*, *y*, *z*)	*p*-value
ReHo analysis
ANOVA among the three groups
Anterior cingulate	L, R	78	18.46	0, 18, −9	^a^*p* < 0.05
TDPD-SD group > TDPD-NSD group
Anterior cingulate	L, R	63	5.22	3, 30, −12	^b^*p* < 0.05
TDPD-SD group > HC group
Anterior cingulate	L, R	6	3.85	3, 30, −9	^b^*p* < 0.05
HC group > TDPD-NSD group
Anterior cingulate	L, R	59	−5.96	0, 18, −9	^b^*p* < 0.05
FC analysis (with the outcome cluster of ReHo analysis as the seed)
ANOVA among the three groups
Olfactory	L	112	11.88	3, 18, −12	^c^*p* < 0.05
Rectus	R
Parietal_Sup	R	105	8.97	18, −54, −57	^c^*p* < 0.05
Precuneus	R
TDPD-SD group > TDPD-NSD group
Rectus	R	19	−4.23	3, 30, −21	^b^*p* < 0.05
HC group > TDPD-SD group
Olfactory	L	9	−2.59	−12, 12, −21	^d^*p* < 0.001
Olfactory	R	4	−2.21	0, 21, −6
HC group > TDPD-NSD group
Olfactory	L	53	4.83	0, 18, −9	^b^*p* < 0.05
Parietal_Sup	R	12	4.20	24, −60, 57
Precuneus	R	14	3.67	9, −48, 57	

*ReHo, regional homogeneity; FC, functional connectivity; TDPD-SD, tremor-dominant Parkinson’s disease with sleep disorder; TDPD-NSD, tremor-dominant Parkinson’s disease with no sleep disorder; HC, healthy controls; R, right; L, left; AAL, automated anatomical labeling; MNI, Montreal Neurological Institute. Differences were considered statistically significant when *p* < 0.05. ^a^ANOVA was performed at a cluster-level threshold of *p* < 0.05 (FWE-corrected) with a cluster-forming threshold at the voxel level *p* < 0.001. ^b^*Post hoc* analysis was performed at a cluster-level threshold of *p* < 0.05 (FWE-corrected) with a cluster-forming threshold at the voxel level *p* < 0.001. ^c^ANOVA was performed at a cluster-level threshold of *p* < 0.05 (FWE-corrected) with a cluster-forming threshold at the voxel level *p* < 0.005. ^d^*Post hoc* analysis was performed at a voxel *p* < 0.001 without correction*.

### FC Analysis Results

Based on ANOVA, significant group effects were observed for FC in the L-OC, R-SG, R-SPG, and right precuneus (*p*-voxel < 0.005, *p*-cluster correction < 0.05).

**Figure 2:**
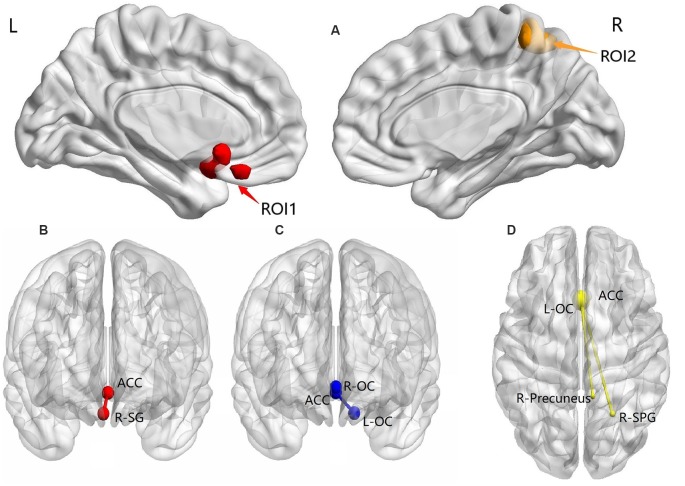
ANOVA and *post hoc* analysis results for FC. **(A)** ANOVA among three groups; **(B)** comparison between the TDPD-NSD and TDPD-SD groups; **(C)** comparison between the HC and TDPD-SD groups; **(D)** comparison between the TDPD-NSD and HC groups. R, right; L, left; ROI1, left olfactory cortex, right straight gyrus; ROI2, right superior parietal gyrus and right precuneus; ACC, anterior cingulate gyrus; SG, straight gyrus; OC, olfactory cortex; SPG, superior parietal gyrus.

*Post hoc* analysis results showed the following: (1) compared with the TDPD-NSD group, TDPD-SD exhibited significantly lower FC values in R-SG with ACC (*p*-voxel < 0.001, *p*-cluster correction < 0.05); (2) compared with the HC group, TDPD-SD exhibited significantly lower FC values in L-OC and R-OC with ACC (*p* < 0.05 uncorrected); and (3) compared with the HC group, the FC of L-OC, R-SPG, and the right precuneus decreased in TDPD-NSDs with ACC (*p*-voxel < 0.001, *p*-cluster correction < 0.05; see Table 2 and Figure 2).

### Correlation Analysis

#### ReHo Analysis

**Figure 3:**
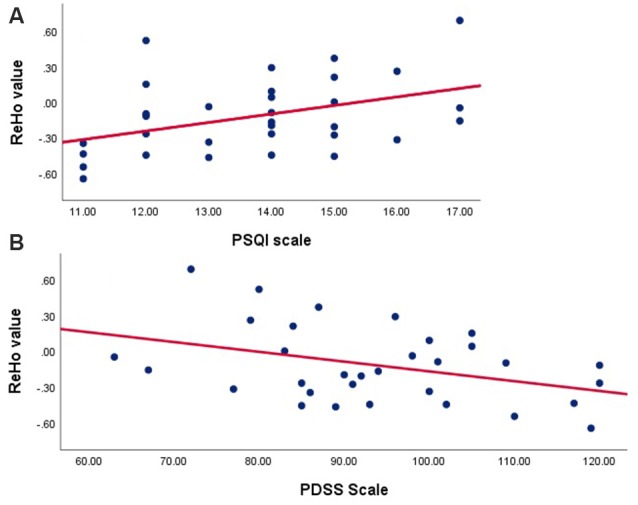
Correlation analyses examining the associations between brain activity with clinical data in the TDPD-SD group. **(A)** Pearson’s correlation analysis between the ReHo value and the PSQI score in the TDPD-SD group; **(B)** Pearson’s correlation analysis between the ReHo value and the PDSS score in the TDPD-SD group. ReHo, regional homogeneity; PSQI, the Pittsburgh Sleep Quality Index; PDSS, Parkinson’s Disease Sleep Scale.

According to outcomes of the Pearson’s correlation analysis, ROI signals extracted in the TDPD-SD group were positively correlated with the PSQI score (*r* = 0.41, *p* = 0.020) but negatively correlated with the PDSS score (*r* = −0.38, *p* = 0.030; Figure 3). There was no correlation between the ROI signals and other clinical data in each group (*p* > 0.05).

#### FC Analysis

There was no correlation between the ROI signals and clinical data in each group (*p* > 0.05).

## Discussion

In this study, we hypothesized that there was alteration in the brain neural mechanism of TDPD-SD which included ReHo/FC features that could be supported by fMRI. Through comparative studies, we found that ReHo values of ACC in the TDPD-SD group had a characteristic increase, which was positively correlated with PSQI scores but negatively correlated with PDSS scores. Second, further FC analysis suggested that there was a significantly different functional connection between the ACC and R-SG. Changes in ReHo values can reflect localized regional brain activities and the synchronization of neural activities between them, while changes in FC can provide a basis for the functional reorganization of the local or entire central nervous system. Our review of the literature indicated that this is the first study to examine a single motor subtype complicated by non-motor symptoms of PD using fMRI and the first to study PD-associated SDs using ReHo and FC analysis. Furthermore, in our ReHo analysis, we found that only one cluster of voxels displayed consistent ReHo; this area included the structures of the anterior cingulate gyrus, right orbitofrontal gyrus, and left caudate nucleus. ReHo analysis revealing only a single cluster of voxels has rarely been reported. Therefore, further seed-based FC and correlation analyses associated with the clinical scale are more targeted. Moreover, the introduction of a healthy group as a control helped us obtain more objective results.

ReHo analysis assesses the status of spontaneous neuronal activity by calculating similarities in fluctuations of the blood oxygen level-dependent signals of adjacent voxels in a voxel cluster (Wu et al., 2009). The abnormality may reflect transient changes in the spontaneous activities of neurons in the local brain region, which means that increased or decreased ReHo values may indicate increases or decreases in the homogeneity of neural activities in local brain regions (Wu et al., 2009). There are two hypotheses for the increased ReHo values in these areas. One hypothesis is the existence of a compensation mechanism in the high ReHo regions of the brain (Luo et al., 2017); however, our results indicated the involvement of only one cluster of voxels (with increased ReHo), with no simultaneous increase or decrease in local ReHo in other regions. Thus, this hypothesis is not supported. The other hypothesis is the overactivation theory, which interprets the increase in local ReHo values as a compensatory mechanism to counteract structural damage in the brain by enhancing the activities of the nerves (Tu et al., 2018). This hypothesis agrees with the results of our correlation analysis; that is, the PSQI scores representing the aggravated sleep disturbances were positively correlated with the ReHo values of ACC. In addition, the negative correlation between PDSS scores and ReHo values of ACC provided further evidence. Therefore, we believe that the increase in ReHo values reflects the aggravation of TDPD-SD and the overactivation of corresponding brain regions. We speculate that the limbic system—predominantly the ACC—is involved in the mechanism underlying SDs in TDPDs, representing a unique neurophysiology and pathological feature.

ACC participates in the regulation of a variety of psychological factors, especially those involved in emotional control, pain stimuli, and cognitive execution skills (Ridderinkhof et al., 2004). A damaged ACC produces many clinical symptoms, including apathy, the inability to pay attention, autonomic nervous system regulation disorders, akinetic mutism, and mood swings (Bush et al., 2000). In recent years, more researchers have attempted to uncover the connections of the limbic system as well as the function of its critical component—the ACC—in SD symptoms in PD patients. However, the number of relevant studies is limited. A study by de Schipper et al. (2017) related to PD and the ACC showed that the absence of or atrophy affecting the integrity of the anterior or posterior cingulate gyrus caused abnormalities that were mainly related to the non-dopaminergic characteristics of the two regional networks, leading to specific symptoms such as cognitive decline and daytime drowsiness. A brain-function ReHo study in patients with PD by Li et al. (2017) suggested that the ACC is involved in regulating fatigue symptoms associated with SDs in Parkinson’s patients. Moreover, some studies linking the contributing roles of the limbic system and the ACC to many PD-related symptoms have also provided evidence supporting our findings. In an RBD-related case report, Erdinc et al. (2009) proposed that the limbic system participates in the mechanism of generating nightmare symptoms in PD patients, thus causing sleep disturbance. This conclusion is supported by Radziunas et al. (2018), with further evidence from PDSD patients; they also found that the white matter in the ACC was reduced in patients with frequent nightmares, suggesting that nightmares are primarily associated with changes in the limbic system and white matter in the frontal lobe. Guo et al. (2018) found that PD-RBD patients exhibited global segmental reorganization and central nerve relocalization in the brain, especially in the limbic system. This discovery provided important guidance for the pathophysiology of PD-associated RBD or other forms of SDs. Based on the above research and our findings, we further speculate that the impact of the ACC on sleep disturbances in TDPD-SDs may originate from its involvement in the regulation of nightmares, its governance of mental and emotional health (such as the induction of depressive symptoms and exacerbation of daytime fatigue that further aggravates SD), or its influence on the pathway of RBD development. Moreover, we speculated that the reduction in ReHo values in TDPD-NSDs was due to the general reduction in ReHo values in ACC in PDs. After a literature review, we found that our conclusion is supported by a previous relevant study (Li et al., 2017), which found decreased ReHo in the ACC in PDs. We thought that the reduction in ReHo values in TDPD-NSDs was due to the general reduction in ReHo values in ACC in PDs, while the increase in ReHo values reflected the aggravation of TDPD-SD and the overactivation of corresponding brain regions to TDPD-related SD. Limbic systems, with ACC as the major cortical component, may play a prominent role in regulating sleep mechanisms in TDPDs.

Furthermore, this study provides new evidence for the functional imbalance of the local network in the TDPD-SD group with seed-based FC analysis. According to the results obtained for the comparison between the two groups (TDPD-SD and TDPD-NSD), we observed a significant difference in FC between ACC and R-SG, which to some extent reflected an imbalance in the local neural function network of TDPD-associated SD. The exact function of SG is still unclear, and there are limited studies on the relationship between rectus and PD/SD. According to our literature review, the results of this study are, to a certain extent, consistent with the results of an idiopathic RBD (iRBD)-related study reported by Han et al. (2019), who found that patients with iRBD had a significantly reduced gray matter volume in the straight gyrus. The results further revealed that FC between ACC and R-SG were significantly increased in the TDPD-SD group, which offered another possibility of an imbalance of the mechanism of TDPD-SD. In addition, the ROIs of *post hoc* analysis in TDPD-SD vs. TDPD-NSD, with R-SG as the main component, should be a small part of the medial prefrontal cortex (mPFC) according to Brodmann’s brain map (Pearce, 2005). It is worth mentioning that the SG participates in and forms a part of the mPFC of the default mode network (DMN), and the mPFC is regarded as an important role of the functional hub of DMN (Raichle, 2015). It is noteworthy that research by Pomares et al. (2019) showed that an abnormal local FC of the mPFC can be observed in idiopathic hypersomnia patients and is strongly associated with subjective daytime sleepiness, which provides a new explanation for the reduced FC of the mPFC (R-SG) in the TDPD-SD group.

Second, L-OC, which demonstrated significant differences in the main effect, showed specific significant differences in the comparison between groups. In fact, some studies (Doty, 2012; Yoon et al., 2019) have shown that the dysfunction of the OC and its related brain metabolic network play a crucial role in monitoring the progression in PD-related iRBD patients, which was consistent with our results, showing an imbalance in local functional connection between ACC and L/R-OC. Although the results further provide evidence for abnormal functional connections between ACC and L-OC, a larger sample size is still needed for statistical correction to provide stronger proof of the outcome. In addition, we found that FC between the ACC and precuneus was reduced in the TDPD-NSD group compared with the HC group, which further supported the conclusion of Thibes et al. (2017) that precuneus-related FC abnormalities are closely correlated with PD motor dysfunction. The precuneus is regarded as the functional core of the DMN and is closely related to some neurological diseases (Matsuoka et al., 2018). Therefore, our results further validate the presence of a DMN functional imbalance in TDPDs.

We speculated that the change in FC in the above structures would lead to an imbalance in brain sleep mechanisms in TDPD-SDs, resulting in damage to the entire sleep network structure in patients. This phenomenon provides another explanation for TDPD-SD pathogenesis. However, correlation analysis between FC analysis and clinical data does not support our hypothesis, which suggests that further research is needed to reach more objective conclusions. The present study has some limitations. First, the sample size was relatively small; this did not affect the results of the ReHo analysis through FWE correction in the ANOVA, but it may have led to a relatively loose correction level in the two-sample *t*-test in the *post hoc* analysis of FC. A larger sample size would allow us to further classify the etiology of the disturbed sleep symptoms in TDPD-SDs and to discover the correlation between the refinement score of the PDSS scale and the ReHo values. It would also allow us to explore the specifically manifested SDs and their related mechanisms and to exclude false-positive results, which are often associated with small sample sizes. Second, we did not compare the results with NTDPDs. Such a comparison may provide a better understanding of the specificities of SDs associated with different motor subtypes of PD. Third, PDs commonly occur in older adults, and the age of the subjects included in this experiment ranged from 50 to 83 years old. Thus, the age span of the included samples was large with a high upper limit. Although age was used as the regression covariate in data processing, the expanded age range may have interfered with the results. Finally, we did not find any common regions that exhibited ReHo alterations in the two patient groups compared with HCs. However, for seed-based FC analysis, we noted that both patient groups exhibited altered ACC FC with L-OC compared with the HC group. The discrepancy may imply differential sensitivities of different methods and/or measures in capturing common and specific brain functional alterations between TDPD-SD and TDPD-NSD.

## Conclusion

Our study demonstrated that the resting brains of TDPD-SDs are characterized by localized increases in ReHo values that reflect an imbalance and hyperactivity in the functional regulation of local brain networks. The limbic system—with the ACC as its main component—is a predominant regulator of the sleep mechanism in TDPDs. The results of this study suggest the presence of a functional reorganization of the central nervous system in response to SDs in TDPD patients, which has not been proven to correlate with clinical data.

## Data Availability Statement

The datasets generated for this study are available on request to the corresponding author.

## Ethics Statement

The studies involving human participants were reviewed and approved by Ethic Committee of Guangdong Provincial Hospital of Chinese Medicine (BF2018-106-01). The patients/participants provided their written informed consent to participate in this study.

## Author Contributions

JC, XLi, and BL conceived and designed the experiments. SZ performed the fMRI scans. XC and XH analyzed the fMRI data. XLu collected and analyzed the clinical data and recruited potential participants. XC and XH wrote the manuscript.

## Conflict of Interest

The authors declare that the research was conducted in the absence of any commercial or financial relationships that could be construed as a potential conflict of interest.

## Footnotes

^1^http://rfmri.org/dpabi

^2^http://www.fil.ion.ucl.ac.uk/spm
